# Changing trends in anaesthesia for trabeculectomy: a clinical effectiveness and safety analysis

**DOI:** 10.1038/s41433-023-02441-y

**Published:** 2023-02-28

**Authors:** P. Asodaria, J. Y. Ng, G. Lascaratos, S. Trikha, A. Kulkarni

**Affiliations:** 1 Faculty of Life Sciences & Medicine, King’s College London, London, UK; 2 Department of Ophthalmology and Vision Sciences, University of Toronto, Toronto, ON Canada; 3 Department of Ophthalmology, King’s College Hospital NHS Foundation Trust, London, UK

**Keywords:** Outcomes research, Surgery

Glaucoma is a progressive optic neuropathy and represents the leading cause of irreversible blindness worldwide [1]. Intraocular pressure (IOP) is an important modifiable risk factor and trabeculectomy remains the procedure of choice for most glaucoma specialists [2, 3]. Anaesthesia choice is paramount when planning for trabeculectomy. Factors to consider include surgeon’s preference, patient’s age and preference, anticipated difficulty and duration of the operation, and logistical concerns [4].

The clinical records of patients who underwent trabeculectomy as a sole procedure in a single tertiary referral trust in London between 2006 and 2022 (King’s College Hospital, London, UK) were retrospectively reviewed using a single electronic medical record system (Medisoft®, Leeds, UK). The criteria for general anaesthesia (GA) in our cohort were very advanced glaucoma at risk of visual field wipe out, only eye, very high IOP and patient preference. All surgeries were performed by a glaucoma consultant or fellow in a standard ophthalmic operating room with anaesthetic consultant cover.

Overall, 26% of trabeculectomies were performed under GA (*n* = 188), 67% under peribulbar local anaesthesia (PLA) (*n* = 490), and 7% under sub-tenon’s local anaesthesia (SLA) (*n* = 51). Our data demonstrate that the use of local anaesthesia (LA) increased substantially from 57% in 2007 to 92% in 2021 (Fig. 1). Mean postoperative VA (LogMAR) was 0.68, 0.74 and 0.52 in GA, PLA and SLA groups respectively (*p* = 0.235). The percentage IOP reduction at final visit compared to IOP at baseline was 32%, 32% and 37% in the GA, PLA and SLA groups, respectively (*p* = 0.111). At 5-years post-trabeculectomy, 49% (*n* = 64), 36% (*n* = 85) and 41% (*n* = 12) required no IOP-lowering medication in the GA, PLA and SLA groups respectively (*p* = 0.168).

**Fig. 1:**
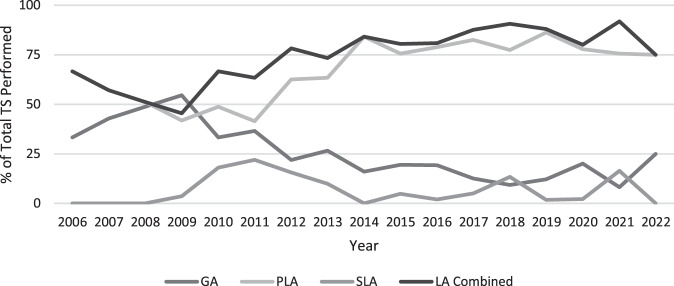
Trends in anaesthetic choice for trabeculectomy surgery (TS) between year 2006 and 2022. PLA (Line 1), LA combined (Line 2), GA (Line 3) and SLA (Line 4). LA combined (PLA + SLA).

Anaesthetic complications were recorded in 9.4% of surgeries performed under LA, 7.8% of PLA cases, and 10% of SLA cases (*p* = 0.607) (Table 1). No anaesthetic complication was documented in the GA group. Post-operative complications occurred in 8% of GA cases, 14.5% of PLA cases and 15.7% of SLA cases (*p* = 0.064) (Table 1). The mean time from first incision to end of procedure was 73 min for the GA group, 81 min for the PLA group and 72 min for the SLA group (*p* < 0.001). Post-hoc testing demonstrated a significant difference between the GA and PLA groups (*p* < 0.001). Longer surgical time in the PLA group may represent the additional time required to ensure adequate anaesthesia and akinesia.

**Table 1:** Raw data regarding of anaesthetic and post-operative outcomes.

	*Complication*	*GA*		*PLA*		*SLA*		*Total*		*p* *value*
		*n*	%	*n*	%	*n*	%	*n*	%	
Post-operative complications	Bleb leak	3	1.6%	27	5.5%	2	3.9%	32	4.4%	0.083
	Choroidal effusion	0	0.0%	1	0.2%	0	0.0%	1	0.1%	-
	Corneal epithelial defect:	1	0.5%	3	0.6%	1	2.0%	5	0.7%	0.516
	Cystoid macular oedema:	0	0.0%	1	0.2%	0	0.0%	1	0.1%	-
	Hyphaema	1	0.5%	3	0.6%	0	0.0%	4	0.5%	-
	Hypotony < 5	8	4.3%	25	5.1%	5	9.8%	38	5.2%	0.281
	Iris to wound	0	0.0%	1	0.2%	0	0.0%	1	0.1%	-
	Post-operative eyelid bruising:	0	0.0%	1	0.2%	0	0.0%	1	0.1%	-
	Post-operative ptosis	0	0.0%	1	0.2%	0	0.0%	1	0.1%	-
	Post-operative uveitis:	1	0.5%	2	0.4%	0	0.0%	3	0.4%	-
	Reduction in vision	0	0.0%	1	0.2%	0	0.0%	1	0.1%	-
	Shallow AC: iris-cornea touch	1	0.5%	3	0.6%	0	0.0%	4	0.5%	-
	Vitreous haemorrhage	0	0.0%	2	0.4%	0	0.0%	2	0.3%	-
	None	173	92.0%	419	85.5%	43	84.3%	635	87.1%	0.064
Anaesthetic complications	Conjunctival chemosis	N/A	N/A	6	1.2%	1	2.0%	7	1.0%	0.658
	Conjunctival chemosis + patient discomfort: moderate	N/A	N/A	1	0.2%	0	0.0%	1	0.1%	-
	Conjunctival chemosis + sub-conjunctival haemorrhage	N/A	N/A	1	0.2%	1	2.0%	2	0.3%	0.491
	Eyelid haemorrhage + bruising	N/A	N/A	1	0.2%	0	0.0%	1	0.1%	-
	Eyelid haemorrhage + bruising + uncontrolled eye movement + patient discomfort: mild	N/A	N/A	1	0.2%	0	0.0%	1	0.1%	-
	Patient discomfort: mild	N/A	N/A	10	2.0%	1	2.0%	11	1.5%	0.969
	Patient discomfort moderate	N/A	N/A	7	1.4%	1	2.0%	8	1.1%	0.764
	Patient discomfort: moderate + uncontrolled eye movement	N/A	N/A	2	0.4%	0	0.0%	2	0.3%	-
	Patient discomfort: severe	N/A	N/A	3	0.6%	0	0.0%	3	0.4%	-
	Patient discomfort: severe + uncontrolled eye movement	N/A	N/A	1	0.2%	0	0.0%	1	0.1%	-
	Sub-conjunctival haemorrhage	N/A	N/A	1	0.2%	1	2.0%	2	0.3%	0.491
	Uncontrolled eye movement	N/A	N/A	4	0.8%	0	0.0%	4	0.5%	-
	None	N/A	N/A	452	92.2%	46	90.2%	686	94.1%	0.697

*N/A* data not available.

Reports on anaesthetic practices for trabeculectomy are sparse. Our findings from this large cohort of patients showed a strong downward trend in the number of trabeculectomies performed under GA and an increase in use of LA over the 16-year period.

A recent study conducted in Australia and New Zealand correlate these findings and reflect the shift towards day case surgery within the NHS [5]. Although PLA is the traditional anaesthetic of choice for trabeculectomy, SLA has gained popularity in recent years, possibly due to the risks of sharp-needle LA. Our data demonstrates that all anaesthetic groups assessed offered similar safety and postoperative outcomes. Future studies to analyse national anaesthetic trends and surgical outcomes, in particular SLA and PLA would be useful.

## Data Availability

The datasets generated during and/or analysed during the current study are available from the corresponding author upon reasonable request.
